# “Colored Physicians” vs. “Negro Doctors”

**Published:** 1886-07

**Authors:** 


### "COLORED PHYSICIANS" VS. "NEGRO DOCTORS."

The *Austin Homœopathic Journal* said a short time ago: "Austin now has three negro doctors." [sic] In the June number of that publication the Editor having suddenly grown very deferential said: "There is to be a Convention of Colored Physicians in Galveston for the purpose of organizing a State Association, they having been refused membership in the Allopathic Association, on account of their color, although perfectly "regular".

This sudden change of tone can only be accounted for by the fact that a very respectable colored physician of this city requested said editor in future to please "spell it with a capital "N" if he *insisted* on calling them "negro doctors."

We assert positively that no colored physician has been refused admission to the State Medical Association; none have applied for membership either there or in the Travis Co. Med. Society. The above was manufactured out of the whole cloth for capital—well, the homœopaths are welcome to all the capital they can make in that way.

The same Journal spoke of Dr. Mary Thompson, having been compelled to resign the office of chairmain of the Section to which she had been elected by the A. M. A. Our interpretation of the affair from reading an account of it in the Journals is that she resigned voluntarily, because one or two individuals had criticised the action of the Section in electing her; but that the Association took any action looking towards bringing about her resignation, we positively deny,—and assign the statement of the h. j. to the same category with the above.

When a convention of physicians was held at Waco to organize the Electro-Therapeutic Association, Dr. Vandavel (colored), was, by resolution, invited to be present. The colored physicians of Austin have a standing invitation to attend the meetings of the Travis County Medical Society and they frequently do so, and are always invited to participate in the discussions; being called on by name in turn, as are the members. We shall be much pleased to see the colored physicians of Texas organize; and we will extend to them the right hand of fellowship, and to each one of them individually who is professionally and morally in good standing. With regard to their making application for membership in any of the Associations of white physicians, or insisting on any "rights," or supposed rights, they have shown a delicacy, and a consideration for the social difference between them and the members of the S. M. A. which would shame a homœopath. Although they outnumber the homœopaths in Texas, they have not "demanded representation" on any "boards", or in any proposed committee on legislation; nor have they "demanded" of the Governor "a share of public patronage;" nor again, at last accounts they had not declared themselves "opposed to the protection of the public health" from all dangers (quacks included.)—Go thou homœopath to the "negro doctor," consider his ways and be wise.

